# Perturbing dimer interactions and allosteric communication modulates the immunosuppressive activity of human galectin-7

**DOI:** 10.1016/j.jbc.2021.101308

**Published:** 2021-10-19

**Authors:** N. T. Hang Pham, Myriam Létourneau, Marlène Fortier, Gabriel Bégin, M. Sameer Al-Abdul-Wahid, Fabrizio Pucci, Benjamin Folch, Marianne Rooman, David Chatenet, Yves St-Pierre, Patrick Lagüe, Charles Calmettes, Nicolas Doucet

**Affiliations:** 1 Centre Armand-Frappier Santé Biotechnologie, Institut National de la Recherche Scientifique (INRS), Université du Québec, Laval, Quebec, Canada; 2 Département de Biochimie, de Microbiologie et de Bio-informatique and Institut de Biologie Intégrative et des Systèmes (IBIS), Université Laval, Québec, Quebec, Canada; 3 PROTEO, the Québec Network for Research on Protein Function, Engineering, and Applications, Université Laval, Québec, Quebec, Canada; 4 Nuclear Magnetic Resonance Centre, University of Guelph, Guelph, Ontario, Canada; 5 Computational Biology and Bioinformatics, Université Libre de Bruxelles, Brussels, Belgium

**Keywords:** galectins, apoptosis, cancer, protein dynamics, allostery, glycobiology, CRD, carbohydrate recognition domain, GAL-7, galectin-7, GBS, glycan-binding site, DIP, dimer interfering peptide

## Abstract

The design of allosteric modulators to control protein function is a key objective in drug discovery programs. Altering functionally essential allosteric residue networks provides unique protein family subtype specificity, minimizes unwanted off-target effects, and helps avert resistance acquisition typically plaguing drugs that target orthosteric sites. In this work, we used protein engineering and dimer interface mutations to positively and negatively modulate the immunosuppressive activity of the proapoptotic human galectin-7 (GAL-7). Using the PoPMuSiC and BeAtMuSiC algorithms, mutational sites and residue identity were computationally probed and predicted to either alter or stabilize the GAL-7 dimer interface. By designing a covalent disulfide bridge between protomers to control homodimer strength and stability, we demonstrate the importance of dimer interface perturbations on the allosteric network bridging the two opposite glycan-binding sites on GAL-7, resulting in control of induced apoptosis in Jurkat T cells. Molecular investigation of G16X GAL-7 variants using X-ray crystallography, biophysical, and computational characterization illuminates residues involved in dimer stability and allosteric communication, along with discrete long-range dynamic behaviors involving loops 1, 3, and 5. We show that perturbing the protein–protein interface between GAL-7 protomers can modulate its biological function, even when the overall structure and ligand-binding affinity remains unaltered. This study highlights new avenues for the design of galectin-specific modulators influencing both glycan-dependent and glycan-independent interactions.

Human galectins (GAL) are oligomeric β-galactosidase-binding lectins assembled from small (∼15 kDa) protomeric carbohydrate recognition domains (CRD). In mammals, they are categorized by their CRD architecture and form three broadly defined structural groups: prototype (GAL-1, -2, -5, -7, -10, -11, -13, -14, -15, -16), tandem repeat (GAL-4, -6, -8, -9, -12), and chimera-type (GAL-3) (1). While prototype galectins exist as noncovalent or disulfide-bridged homodimers, tandem repeat galectins are built from heterodimeric CRDs covalently linked by short peptide linkers. In contrast, the monomeric chimera-type GAL-3 is unique in its ability to oligomerize through its collagen-like N-terminal tail (2). Galectins are known to bind cell surface glycoconjugates *via* their glycan-binding sites (GBS), initiating the formation of an extracellular lattice through divalent and multivalent cross-linking of glycosylated receptors (3). This dynamic lattice imparts galectins with the ability to regulate multiple cellular functions, including cell adhesion, cell signaling, and intracellular trafficking (4). This is particularly true when galectins bind to glycoreceptors on activated immune cells to induce apoptosis (5). As a result, galectins act as key apoptotic regulators and potential disease targets in multiple disorders, including cancer tumor progression and metastasis (6).

Among prototype galectins, galectin-7 (GAL-7) is recognized for its preferential expression profiles in normal epithelial cells (7). When overexpressed in many tissues, it can accelerate cancer progression. This is particularly true for lymphoma (8, 9), triple-negative breast cancer (10, 11), endometrial cancer (12), and other subtypes of cancer (13). GAL-7 also plays important cellular functions in cell adhesion, migration, differentiation, proliferation, and apoptosis (13) *via* glycan-dependent or glycan-independent protein–protein interactions with other cellular partners. For example, while extracellular GAL-7 can trigger apoptosis of activated T cells following binding to glycoreceptors *via* its GBS (14), it can also bind E-cadherin on epithelial cells independently of its GBS. Glycan-independent interactions implicating GAL-7 have also been reported inside the cells, most notably with the antiapoptotic BCL-2 regulator (15). Overexpression of GAL-7 has also been implicated in other pathologies, including preeclampsia (16) and abnormal wound healing of the skin and cornea (17, 18).

For more than a decade, the development of galectin modulators has almost exclusively focused on sugar-based, small-molecule compounds aimed at perturbing glycoreceptor interactions (19). However, the high degree of GBS homology among family members renders highly specific, high-affinity galectin modulators extremely difficult to synthesize. As a result, GBS inhibition remains a high-risk strategy because of unwanted off-target effects involving binding to other highly homologous and often beneficial antitumoral galectin members, *e.g.*, GAL-4 (20, 21). To further complicate matters, an increasing number of studies have now confirmed the importance of GBS-independent activities modulated by galectins (22, 23, 24), including potentially relevant hetero-oligomeric galectin architectures, modular designs, and valence variability (25, 26, 27, 28). This should come as no surprise, as it has been known for a while that lectins can bind noncarbohydrate compounds, often exhibiting higher affinities than their “natural” saccharide ligands (29). GBS inhibitors are ineffective at targeting glycan-independent galectin function, further exemplifying the need to establish new approaches for targeting unique galectin members in highly specific therapeutic circumstances.

These observations have awakened interest in targeting and modulating galectin function using newly developed allosteric effectors. In many instances, allosteric modulation of protein function was shown to be more selective and effective than traditional orthosteric inhibition (30). Furthermore, such strategy has proven effective in finding compounds inhibiting mammalian C-type lectins, a protein family initially deemed undruggable (31, 32). Targeting non-GBS regions in galectins would also offer means to develop new generations of galectin inhibitors that specifically modulate glycan-independent functions in the cell, a therapeutic strategy that remains marginally represented. In support of this avenue, galectins have been shown to undergo evolutionary pressure that stabilizes their quaternary oligomeric architecture to improve ligand affinity and biological function (33). The relatively low sequence identity and unique dimer architecture among members of the prototypic galectin family (3) offer means to specifically target their dimer interface to improve inhibitor specificity.

We recently developed GAL-7 dimer interfering peptides (DIPs) to alter dimer stability in this functionally important protein (34). Among selected designer sequences, peptide hGAL-7(129–135) was shown to effectively reduce the proapoptotic activity of GAL-7 on Jurkat T cells by disrupting the monomer–dimer equilibrium in solution. This sequence was also shown to promote accumulation of GAL-7 on the surface of T cells. These results suggest that dimer interface perturbation might alter the specificity and affinity of the GAL-7 GBS against distinct glycosylated receptors, potentially acting *via* an allosteric mechanism involving homodimer interface communication. This hypothesis is further strengthened by prior work suggesting the existence of lactose-induced, long-range positive cooperativity between the two GBSs on opposite GAL-7 protomers (35). Despite being largely uncharacterized, positive cooperativity behavior suggests the involvement of long-range, organized residue networks relaying dynamic information between GAL-7 protomers. Further, characterizing the relationship between the biological function of GAL-7 and allosteric communication would significantly improve our ability to design GAL-7-specific allosteric inhibitors.

In this work, we studied the impact of homodimer interface mutations on the induction of Jurkat T cell apoptosis, allowing us to positively and negatively modulate the biological activity of GAL-7 by designing a covalent disulfide bridge (G16C) and destabilizing mutation (G16S) to control homodimer strength, stability, and biological activity. Biophysical, structural, and computational characterization of G16X variants provides a clearer view of the allosteric network governing molecular function in GAL-7.

## Results and discussion

### Prediction and design of GAL-7 variants that destabilize homodimer integrity

A number of structural studies have previously highlighted the unique “back-to-back” homodimer architecture adopted by GAL-7 in solution (3, 33, 36, 37). Some reports have also alluded to the potential importance of interface residues involved in dimer formation and stability, in addition to proposing the existence of allosteric networks connecting the two distant GBS sites on opposite GAL-7 protomers (35). To confirm complex formation and stability in apo and holo forms, we tested the integrity of the GAL-7 homodimer in solution. NMR translational diffusion measurements were performed on free and lactose-bound WT GAL-7 complexes of increasing protein–ligand molar ratios. Our results not only confirm the existence of a stable WT GAL-7 homodimer in solution, but further demonstrate that diffusion coefficients are not significantly altered upon addition of increasing lactose concentrations to GAL-7 (Fig. S1). This supports structural integrity, stability, and biological relevance of a stable WT GAL-7 homodimer in its apo and holo forms.

The propensity of GAL-7 to maintain homodimer integrity upon interface perturbation was thus interrogated by performing computational mutational predictions at the interface using the algorithms PoPMuSiC (38) and BeAtMuSiC (39). These tools provide computer-aided design of all possible single-site mutational replacements in proteins. PoPMuSiC evaluates the folding free energy changes (ΔΔG_F_) resulting from each mutated site, while BeAtMuSiC evaluates protein–protein binding free energy alterations upon mutation (ΔΔG_B_). Both algorithms were used in complementary fashion to help with prediction and design of experimental point mutations that effectively promote stabilization or destabilization of the GAL-7 monomer–dimer equilibrium.

We first searched for GAL-7 protomer interface mutations that favored stabilization of the monomer over that of the dimer. Using PoPMuSiC, we computationally introduced and evaluated all possible single-site mutations in GAL-7. Residues located at the dimer interface and exhibiting solvent accessibility differences greater than 10% between monomer and dimer states were prioritized. Mutations with significant monomer–dimer stability differences were selected, as defined by ΔΔG_F_ (dimer) - ΔΔG_F_ (monomer) ≥ 2 kcal/mol (Table S1). Amino acid replacements satisfying these criteria were found at positions Gly16, Val18, Ile91, and Phe135 (Table S1). Mutations at positions Gly16/Phe135 were prioritized over Val18/Ile91 since they exerted a greater number of dimer destabilizing effects in addition to displaying an extended dimer interface, as exemplified by greater solvent accessibility changes upon binding. We applied further restrictions on the variants to prevent detrimental secondary structure perturbations or disulfide bridge formation (*i.e.*, no Gly, Pro, Cys replacements) (Table S1). We also avoided variants that altered the overall charge of the protein. BeATMuSiC calculations predicted significant dimer affinity alterations for all remaining variants, with ΔΔG_B_ ≥ 4 kcal/mol (Table S1). We finally prioritized individual substitutions G16S and F135S, as they caused the largest ΔΔG_B_ among all remaining variants. These two variants were thus experimentally tested in the context of GAL-7 dimer stability and function. Interestingly, the main chain oxygen atoms of Gly16 and Phe135 both make inter-protein H-bonding interactions with the Nζ atom of Lys98, according to the Protein Interaction Calculator (PIC) (40). Phe135 is also involved in hydrophobic contacts with Leu89, Ile91, and Val100 on the opposite chain. We expected that mutation to serine would break these interactions.

As a counterpart to destabilizing mutations, we also searched for mutational predictions that favored stabilization of the GAL-7 homodimer rather than its monomeric form. Analysis of the WT homodimer structure (PDB entry 4GAL) highlighted ideal distance between Gly16-Cα atoms in each protomer (4 Å), suggesting that introduction of a cysteine at this site could favor formation of a covalently linked GAL-7 homodimer through formation of a disulfide bridge, with only slight structural reorganization. As a result, we also designed a G16C variant for further functional and structural investigation.

Based on our computational predictions, mutations G16S and F135S should weaken GAL-7 homodimer interactions, while formation of a disulfide bridge in G16C could strengthen protomer interactions and favor GAL-7 homodimer stability. Recombinant expression of mutational constructs yielded soluble proteins for the G16X variants, but F135S was found to be systematically expressed as inclusion bodies, despite several trials to improve its solubility. These results suggest irreversible structural alterations and/or limited stability upon introduction of a polar residue at position 135. Interestingly, Phe135 is the terminal residue within the primary structure of GAL-7, forming van der Waals interactions with neighboring residues Ile91, Lys98, and Asp103 on β-strand 7. A ConSurf analysis (41) illustrates that this position is the terminal amino acid residue for only 12/81 nonredundant galectin homologs, exhibiting limited sequence variability and strict hydrophobic conservation (Phe, Val, Leu, and Ile). This observation suggests that replacing the benzyl moiety with a polar hydroxyl group at position 135 impedes essential hydrophobic interactions involved in preserving monomer–dimer stability in GAL-7.

### Perturbing homodimer stability alters the proapoptotic activity of GAL-7

**Figure 1:**
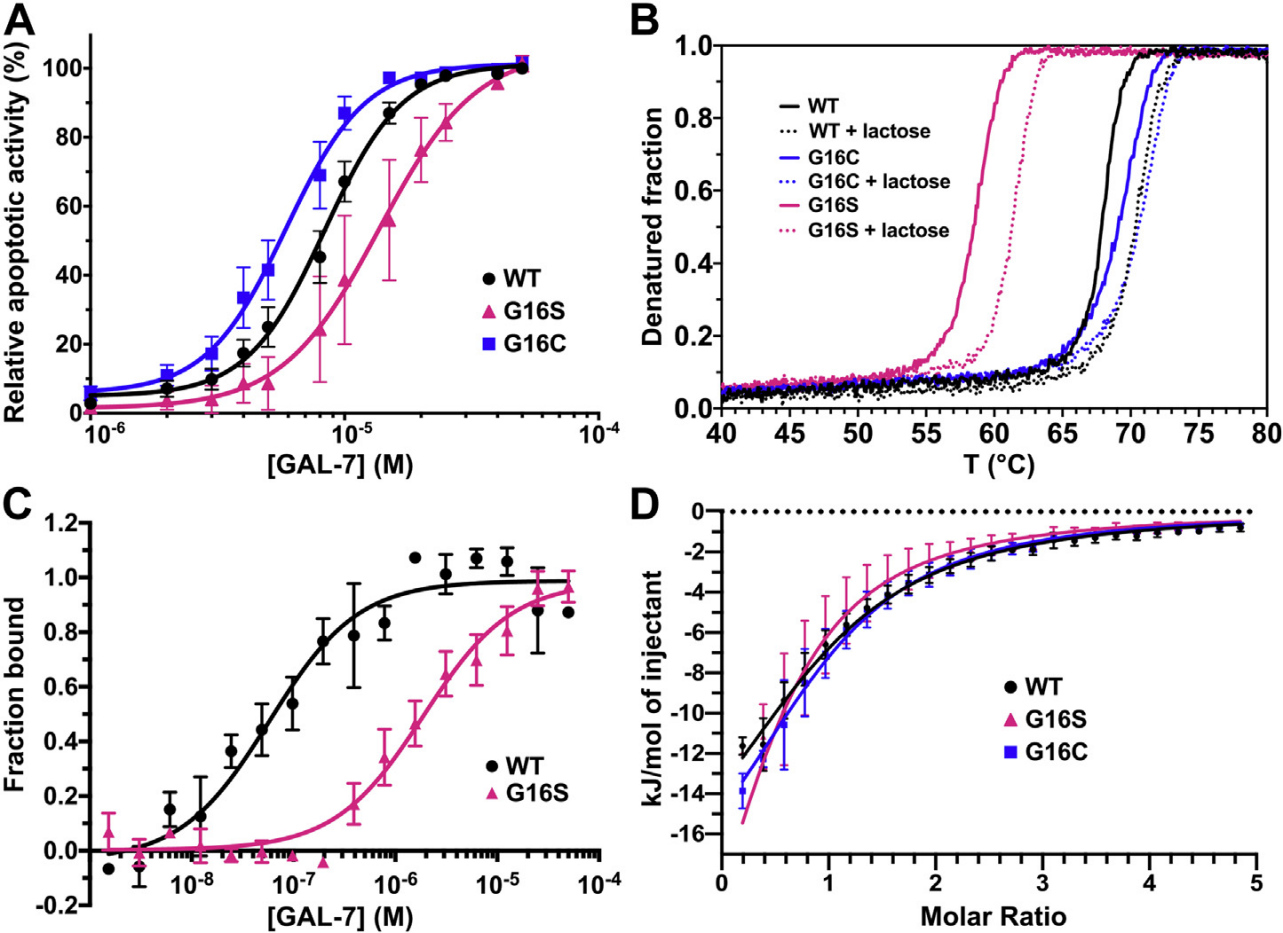
**Single-site dimer-interfering mutations G16C and G16S act as positive and negative functional regulators of the proapoptotic activity of GAL-7.***A*, GAL-7-induced apoptosis of human Jurkat T cells for WT GAL-7 (*black circles*), G16S (*pink triangles*), and G16C (*blue squares*), as evaluated by positive Annexin V staining using flow cytometry analysis. *B*, thermal stability of WT GAL-7 and variants G16S and G16C in the absence and presence of lactose, as measured by CD-induced thermal denaturation. *C*, dimer equilibrium affinity of WT GAL-7 and the G16S variant, as measured by MST. *D*, α-lactose-binding isotherm of WT GAL-7 and variants G16S and G16C, as measured by ITC.

Galectins are known to induce apoptosis of human T cells by binding to their glycosylated receptors, thereby modulating cell fate in diseases such as cancer (42). For a number of years, our group has extensively used GAL-7 as a relevant model for studying Jurkat T cell induced apoptosis, providing additional information on molecular and cellular mechanisms governing GAL-7 function in the cell (11, 14, 34, 43). Despite several studies detailing the existence of a homodimeric structure in GAL-7, few reports have thus far interrogated the importance of maintaining the integrity and stability of this dimer for preservation of function. To investigate the computational predictions of G16X replacements, we performed Jurkat T cell apoptosis experiments with variants G16S and G16C. Our results show that the G16S mutation decreases the proapoptotic activity of GAL-7, yielding an EC50 of 13.7 μM (95% confidence interval [CI95%] between 10.2 and 18.3 μM) relative to 8.4 μM (CI95% 7.6–9.1 μM) for WT GAL-7 (Fig. 1*A*). Conversely, the G16C variant has a greater capacity to induce apoptosis of Jurkat T cells than WT (Fig. 1*A*), yielding an EC50 of 5.9 μM (CI95% 5.2–6.7 μM). These results suggest that residue Gly16 is directly involved in monomer–dimer stabilization and/or allosteric communication between protomers in GAL-7.

### Perturbing dimer interface alters GAL-7 stability but does not affect glycan-binding affinity

The overall fold and stability of the GAL-7 variants were assessed by performing CD spectropolarimetry in the presence and absence of *α*-lactose. Under these conditions, the far UV molar ellipticity spectra (200–260 nm) of all proteins is virtually indistinguishable, further illustrating that Gly16 mutations do not perturb the overall fold of free or lactose-bound GAL-7 (Fig. S2). Thermal denaturation experiments were also carried out to examine the effects of mutations on GAL-7 stability. CD melting curves show that G16C is the more stable variant (T_m_ = 70.0 ± 0.1 °C), followed by WT (T_m_ = 67.8 ± 0.2 °C) and G16S (T_m_ = 58.7 ± 0.2 °C) (Fig. 1*B*). These results confirm the thermal stability advantage conferred by the G16C mutation, which provides a 2.2 °C increase in T_m_ relative to WT. Conversely, the G16S mutation weakens GAL-7 stability, inducing a 9.1 °C decrease in melting temperature. By incubating protein with saturating concentrations of *α*-lactose, we observe an overall thermal stability increase of 2.7 °C for WT (T_m_ = 70.5 ± 0.1 °C), 1.9 °C for G16C (T_m_ = 71.9 ± 0.2 °C), and 2.9 °C for G16S (T_m_ = 61.6 ± 0.1 °C). These results confirm the previously observed thermal stability advantage conferred by lactose binding to GAL-7 (35). This effect is more prominent in WT GAL-7 and variant G16S than in G16C, further suggesting the existence of a covalent link in the latter variant.

We also used microscale thermophoresis (MST) to investigate the strength of dimer association and equilibrium induced by Gly16 replacements. In this experiment, the fluorophore-labeled GAL-7 dimer is incubated at higher temperature with unlabeled GAL-7, resulting in weakening of noncovalent dimer interactions and induction of equilibrium exchange between labeled and unlabeled complexes to form mixed heterodimers. This allows extraction of the GAL-7 equilibrium dimer dissociation constant (*K*_D_) under specific experimental conditions and protein concentration range. It also further provides an estimate of dimer affinity perturbations induced upon mutation at the protomer interface. GAL-7 was previously shown to be predominantly dimeric at concentrations around 1.6 μM and above (35). Consistent with these observations, our MST results show that WT GAL-7 adopts a dimeric form in similar experimental conditions, exhibiting a dimer equilibrium dissociation constant (*K*_D_) of 0.06 μM (Fig. 1*C*). In contrast, the G16S variant displays 32-fold lower dimer affinity relative to WT GAL-7 (*K*_D_ = 1.88 μM), indicative of significant homodimer destabilization induced by the mutation. As hypothesized, no binding-associated MST signal was observed for G16C, lending support to stabilization by formation of a disulfide bridge between the two G16C GAL-7 protomers. The dimer interaction energies between the apo WT, G16C, and G16S were also evaluated using FoldX (44) and protein structures from MD simulations (see below). Results were averaged over 5000 dimer structures from the respective trajectories of WT and G16X variants. Consistent with our MST results, G16S was found to be the least stable homodimer, with a dimer interaction energy of -11.6 ± 0.1 kcal/mol. Interaction energies of −13.1 ± 0.2 kcal/mol and −14.8 ± 0.1 kcal/mol were also calculated for WT and G16C, respectively. As expected, the disulfide bridge between the G16C homodimers was found to be a significant contributor to dimer stability.

In addition to testing dimer stability, we also performed ITC experiments to investigate whether mutations at the dimer interface affect long-range glycan-binding affinity in the GAL-7 GBS. Our results show that α-lactose-binding affinities (*K*_D_) were found to be similar for WT GAL-7 and G16X variants (Fig. 1*D* and Table S2). Closer thermodynamic investigation illustrates that although WT and G16C exhibit very similar entropic (Δ*S*) and enthalpic (Δ*H*) contributions to ligand binding, variant G16S shows significantly altered Δ*H* and Δ*S* contributions relative to the two more stable WT and G16C forms of the protein, in line with the lower stability observed in variant G16S.

### G16C and G16S variants maintain dimer architecture in free and lactose-bound states

**Figure 2:**
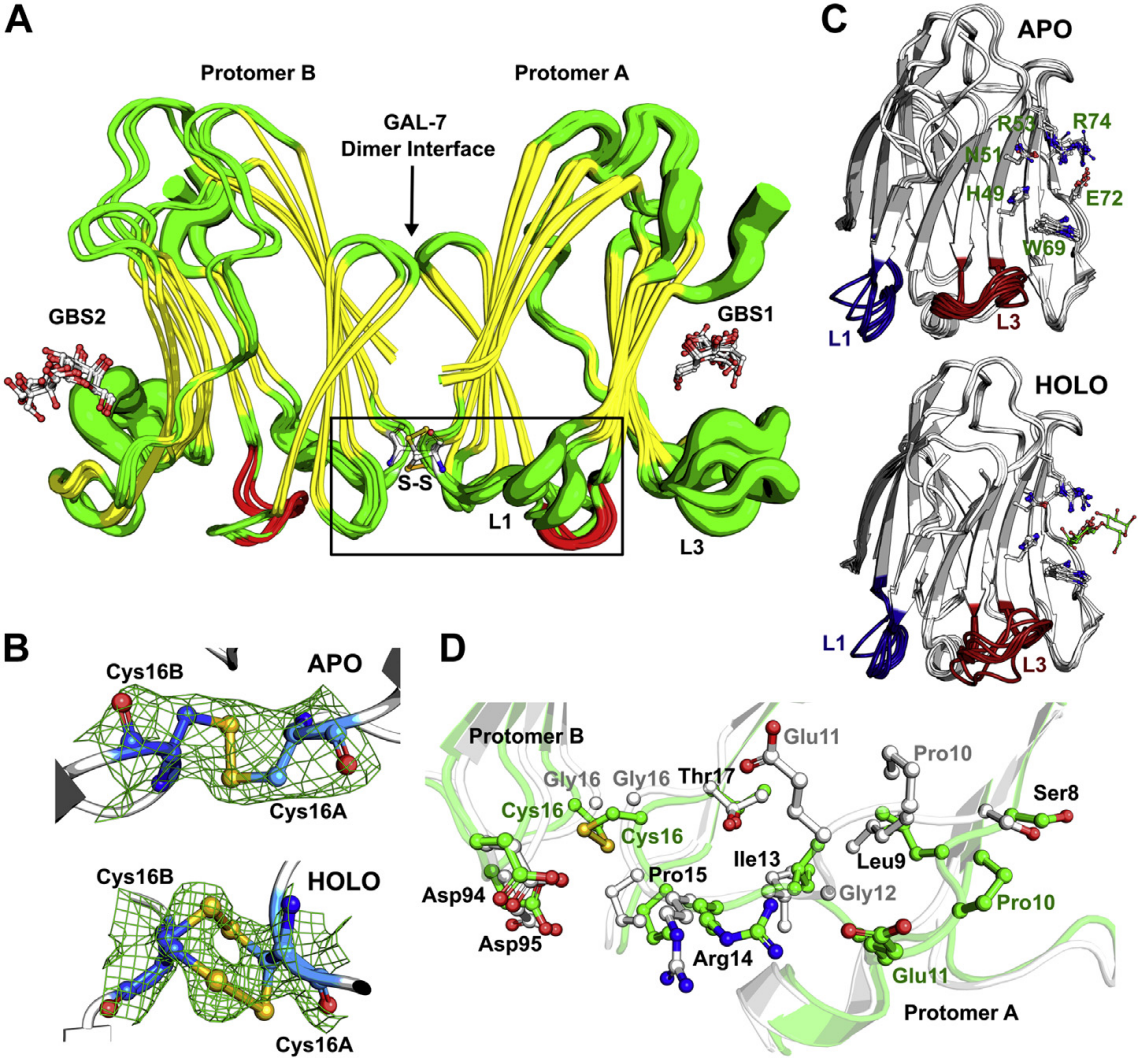
**Crystal structures of WT, G16S, and G16C variants of GAL-7.***A*, structural overlay of ligand-bound forms of WT GAL-7 (PDB 4GAL and 6VTO), G16S (PDB 6VTS), and G16C (PDB 6VTQ). Width of putty cartoon representation illustrates B-factor values, highlighting conformational variations observed in loop 1 (L1) and loop 3 (L3). The engineered disulfide bridge between protomers A (*right*) and B (*left*) in variant G16C is labeled S-S and shown in ball-and-stick representation. The two opposite glycan-binding sites are labeled GBS1 (protomer A) and GBS2 (protomer B). Bound ligands are shown in red-and-white atomic representation. Local environment perturbations resulting from G16X mutations in L1 are shown in *panel D* (*black rectangle*). *B*, electron density map surrounding the Cys16A-Cys16B disulfide bridge at the dimer interface of apo (*top*) and holo (*bottom*) GAL-7 variant G16C. *C*, overlay of CRD protomers A and B in apo (*top*) and holo (*bottom*) structures of WT GAL-7 and variants G16S and G16C. L1 (*blue*) and L3 (*red*) are highlighted in apo (PDB 1BKZ, 3ZXF, 6VTP, and 6VTR) and holo (PDB 4GAL, 6VTO, 6VTQ, and 6VTS) GAL-7 structures. Main GBS residues are labeled and shown in ball-and-stick representation on apo panel. *D*, atomic view of the overlay between WT (*white*) and G16C variant (*green*) showing the local environment surrounding the Gly16 site of mutation and neighboring conformational change experienced by selected residues in loop 1.

Since the proapoptotic activity of GAL-7 on Jurkat T cells involves glycosylated receptors and since no significant change in lactose-binding affinity was observed in variants G16C and G16S, it is unclear how homodimer formation and stability modulate GAL-7 activity in the cell. To examine whether these functional changes are rooted in structural perturbations at the molecular level, we solved the X-ray structures of lactose-bound WT GAL-7 and that of variants G16C and G16S in their apo and holo states (Table S3). We found that both G16C and G16S variants maintain GAL-7 dimer architecture in solution (Fig. 2*A*), unlike other protomer interface mutations that likely perturb the hydrophobic core of the dimer interface, resulting in insoluble constructs (*e.g.*, F135S). Apo structures of G16C (PDB 6VTP) and G16S (PDB 6VTR) were crystallized as dimers in the *P*2_1_2_1_2_1_ space group at 2.3 Å resolution (Table S3). As predicted from our calculations and in support of our MST results, the apo G16C omit map revealed the formation of a Cys16-Cys16 disulfide bridge at the dimer interface (Fig. 2*B*). Overall, GAL-7 dimer architecture is minimally perturbed, as illustrated by Cα structural alignments between apo WT GAL-7 (PDB 3ZXF) and apo G16C (RMSD 6VTP *versus* 3ZXF = 0.686 Å) or between apo WT and apo G16S (RMSD 6VTR *versus* 3ZXF = 0.629 Å). The lactose-bound holo crystal structures of WT (PDB 6VTO), G16C (PDB 6VTQ), and G16S (PDB 6VTS) were resolved at 1.69 Å, 1.95 Å, and 1.9 Å, respectively. These structures also appear as dimers in the *P*2_1_2_1_2_1_ space group (Table S3 and Fig. 2*A*). Similar to the apo forms, no major change in the overall structure was observed between WT GAL-7 and G16C (RMSD 6VTQ *versus* 6VTO = 0.477 Å) or between WT and G16S (RMSD 6VTS *versus* 6VTO = 0.357 Å). Much like its apo counterpart, a Cys16-Cys16 disulfide bridge between each protomer was also confirmed by the presence of a clear electron density map in the G16C holo structure (PDB 6VTQ). In contrast to the apo G16C structure, two disulfide bridge conformers are observed in the electron density of the Cys16-Cys16 covalent bond at the dimer interface of holo G16C (Fig. 2*B*).

### Glycan-binding site organization and ligand positioning

In accordance with the overall structural similarity, overall GBS organization and residue positioning remain largely unchanged between WT and G16X variants. This was expected since Gly16 mutations are located at the homodimer interface, more than 20 Å away from the GBS. Except for Arg71, side chain conformations for all GBS residues were found to adopt similar orientations in all GAL-7 holo structures (Fig. 2*C*). However, since Arg71 is located at the crystal contact surface, this dissimilarity could easily be an artifact of crystal packing. Lactose positioning within the GBS also remains analogous for WT GAL-7 and G16X variants, preserving the vast majority of the previously described polar interactions (37). These results are supported by the largely unaffected α-lactose-binding affinities (*K*_D_) calculated for WT GAL-7 and G16X variants (see above). The previously published lactose-bound WT GAL-7 structure of Leonidas *et al.* (37) (PDB 4GAL) was shown to limit lactose access to the binding site of one GAL-7 protomer due to crystal packing. In contrast, our omit maps clearly show the presence of a bound lactose molecule in the GBS of both protomers within the WT, G16C, and G16S complexes (Fig. 2*A*). This is likely explained by the use of different crystallization techniques, *i.e.*, soaking of WT GAL-7 crystals in lactose solution (37) *versus* cocrystallization with lactose (present study). Interestingly, the D-glucose moiety of lactose was found to adopt an open linear chain configuration in our WT GAL-7 structure (PDB 6VTO), contrary to its typical closed pyranose ring. This could result from X-ray irradiation during crystal shooting (45) or protonation of the ring oxygen atom of the glucose moiety by the nearby terminal guanidinium group of Arg74. In a similar fashion, the ring opening of glucose was previously shown to be catalyzed by the amine group of a nearby lysine in the binding site of human serum albumin (46).

### Apo and holo GAL-7 structures suggest distinct dynamic behavior for loops 1, 3, and 5 in G16X variants

Previous simulations suggested the existence of positive binding cooperativity in GAL-7, whereby entropic penalty at a ligand-free binding site (*i.e.*, increased dynamics in GBS residues) may facilitate induced fit and binding of a second ligand to the GBS in the opposite protomer (35). It has been proposed that these effects would be compensated by rigidification of other internal motions observed elsewhere in the protein. In line with this observation and despite overall structural similarity to WT GAL-7, apo *versus* holo G16X structures suggest distinct long-range conformational alterations triggered by interface mutation and ligand binding. One of the most significant structural rearrangements between WT and G16X apo structures occurs in the local environment of residues 8–17 (loop 1), which exhibit significant atomic-scale deviations in the variants (Fig. 2, *A*–*C*). This local rearrangement of loop 1 involves Gly16 and its neighboring residues, particularly residues Pro10, Glu11, Gly12, and Arg14 (Fig. 2*D*). Contrary to apo structures, the conformation of loop 1 in both chains is not as significantly perturbed in ligand-bound WT and G16X variants (Fig. 2*C*).

To evaluate conformational changes observed between apo and holo structures independent of X-ray artifacts and crystal variability, Z-scores of the atomic B-factors were calculated and used for comparative assessment (47). Cα B′-factors between apo and holo WT structures show that chain B is less flexible, while chain A is more flexible in the presence of lactose (Fig. S3*A*). This indicates that loop 1 experiences distinct dynamic behavior in each GAL-7 chain upon ligand binding, an observation that was not immediately obvious from a previous GAL-7 dynamic investigation (35). Similarly, although both chains in G16X structures exhibit increased Cα B′-factor values for residues 10–15 (loop 1), the mobility gain in one chain was found to be significantly higher than that observed in the opposite chain (Fig. S3, *B* and *C*). In addition to the previously highlighted holo loop 1 rigidification in WT (35), these results suggest that GAL-7 may rely on an asymmetric allosteric network involving distinct loop 1 rigidification (or flexibility) in chains A and B to facilitate cooperativity between the two protomers. Even when similar loop 1 conformations are observed in each chain between holo WT and G16X structures (Fig. S3, *D* and *E*), Cα B′-factor analysis shows an increase in loop 1 mobility for the mutationally induced G16X structures upon ligand binding. These observations further suggest the importance of loop 1 dynamics in interface communication between protomers, in line with the proapoptotic functional effects we illustrated above.

In comparison with holo WT, holo G16X structures also exhibit higher Cα B′-factor values in loop 1, especially for residues Ile13, Arg14, and Pro15, which are located near the site of mutation. In some protomer–protomer interactions, a shift in the side chain of Arg14 leads to the loss of a salt bridge between its terminal guanidinium moiety and residue Asp94 on the opposite protomer. Neighboring residues Asp94 and Asp95 on the opposite protomer also exhibit altered conformational states relative to WT. This leads to the loss of a salt bridge between Arg14 and Asp95 in the G16X variants, a result supported by reduced population of this electrostatic interaction in our MD simulations (see below). These results suggest that homodimer destabilization in variant G16S is partly attributed to changes in side chain conformation and dynamics involving residues Arg14, Asp94, and Asp95, which also neighbor the site of mutation (Fig. S3, *D* and *E*). This structural reorganization also results in the overall reduction of the surface area defining the dimer interface in G16C and G16S variants (Table S4).

Besides loop 1, holo structures of G16S and G16C exhibit increased conformational variations in residues 37–46 (loop 3) relative to WT, a structural element neighboring the GBS (Fig. 2*C*). The Cα B′-factor of these residues increases in the presence of lactose for WT and G16X variants (Fig. S3, *A–C*). However, except for Glu41, G16X variants exhibit higher loop 3 Cα B′-factor values than WT (Fig. S3, *D* and *E*). Moreover, holo structures of WT and G16X variants display distinct rigidity behaviors in residues 64–74 (loop 5), a structural element encompassing several GBS residues. In the presence of lactose, loop 5 Cα B′-factor values decrease in WT, while conversely increasing in G16X variants (Fig. S3, *A–C*). Increased loop 3 dynamics upon ligand binding supports the importance of long-range allosteric communication between the dimer interface, the GBS, and neighboring structural elements. Furthermore, although gain of loop 5 dynamics for G16X variants does not significantly contribute to the affinity of small glycan compounds such as lactose, it might still affect GAL-7 binding to more complex glycoreceptors.

### G16X variants experience similar residue fluctuations but altered interprotomer dynamics relative to WT

To further investigate the role of interface mutation on potential allosteric communication in GAL-7, we performed principal component analysis (PCA) to allow visualization of the overall protein dynamics. The Cα backbone root mean square fluctuations (RMSFs) for each residue within apo WT, G16C, and G16S are presented in Figure S4, while the first five PCA normal modes are presented in Figure S5. Movies WT-PCA.mov, G16C-PCA.mov, and G16S-PCA.mov are also presented in supplementary information for the first ten PCA normal modes of WT, G16C, and G16S, respectively. For all GAL-7 variants, most residues display RMSF values below 1.5 Å, suggesting overall protein rigidity, except for specific segments. In addition to the N-terminus, protein segments exhibiting significant mobility (*i.e.*, higher RMSF values) are located in residues 8–12 (located in loop 1), 39–43 (in loop 3), and 64–68 (in loop 5). For both protomers and for all three protein systems, the highest mobility observed lies within residues 64–68 (loop 5). Noteworthy, the C-termini of GAL-7 displayed low RMSF values because of its location at the homodimer interface. Overall, we observe no significant difference in RMSF values between WT and G16X variants. However, comparison of apo WT with apo G16X normal modes supports the involvement of long-range, global alterations to the rocking movement between protomers triggered by the mutations at the interface (Fig. S5).

### GAL-7 dynamical network analysis uncovers critical edges that define interprotomer communication between the two glycan-binding sites

A previous study observed positive cooperativity in ligand binding to GAL-7 (35), suggesting that one or more long-range allosteric residue networks can modulate binding properties between the two opposite glycan-binding sites in the GAL-7 homodimer. As described previously (48), we used a dynamical network analysis approach similar to the dynamical network of residue–residue contact to calculate allosteric effects in a protein (49). This network analysis was performed on WT GAL-7 and variants G16X to identify potential allosteric pathways that connect the GBS within each protomer and to estimate the effect of mutation on network pathways. Details of network construction and allosteric pathway identification are described in the Experimental procedures.

**Figure 3:**
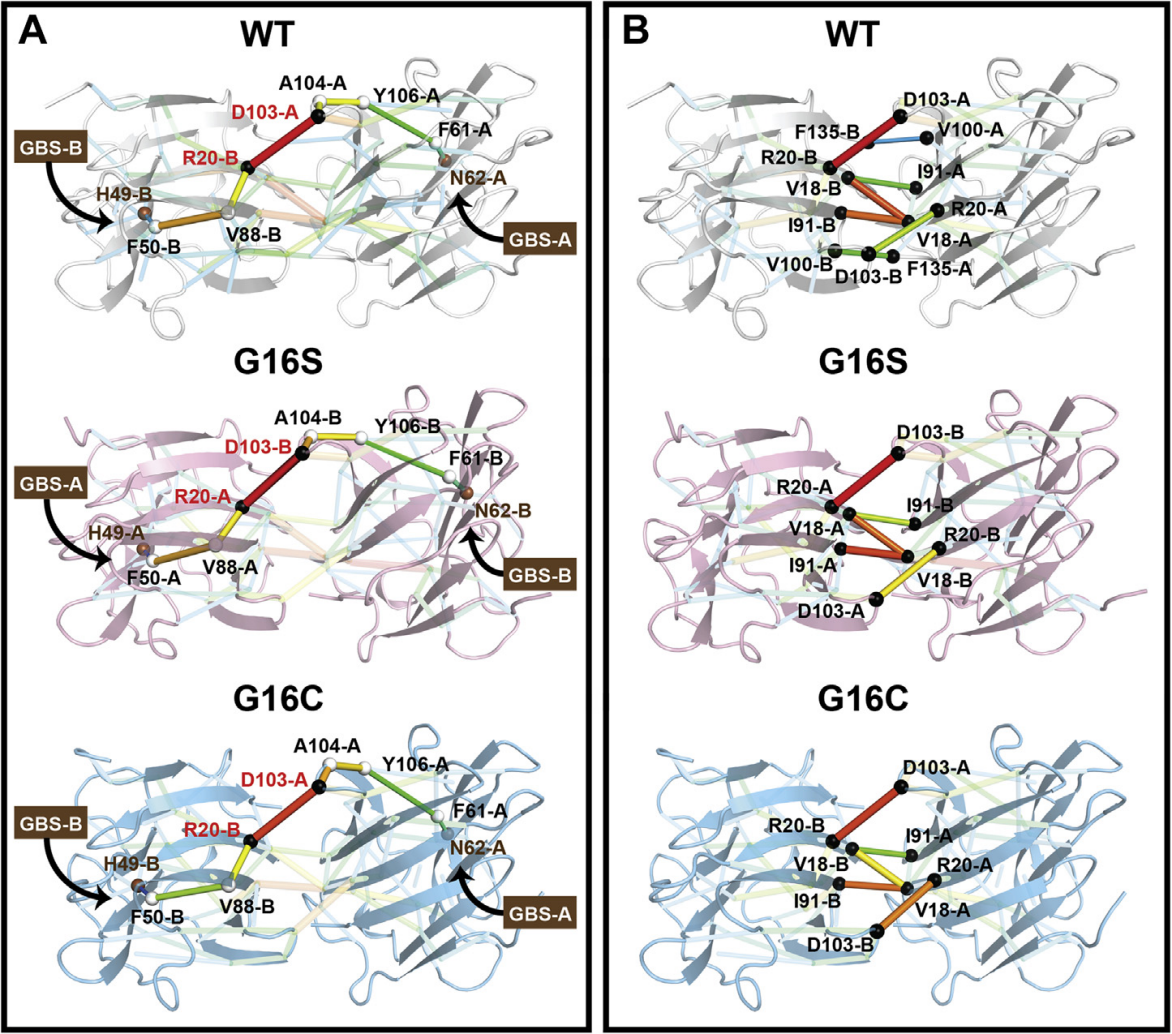
**Dynamical network analysis of WT GAL-7, G16S, and G16C variants.***A*, the shortest pathway between two opposite GBSs (N62-H49) is highlighted on WT GAL-7 (*white*, *top*), G16S (*pink*, *middle*), and G16C (*blue*, *bottom*). Residues involved in interprotomer communication at the dimer interface are represented by *black spheres*. Communication between residues is represented by *sticks*. The critical edge of communication is color scaled from *blue-to-red* and is proportional to stick thickness. Selected GBS residues are labeled and colored *brown*. *B*, residues involved in interprotomer communication at the dimer interface are represented by *black spheres*, with similar critical edge color scale and thickness to represent importance. For clarity and better visual comparison, note that protomers A and B are swapped in variant G16S.

Our results illuminate critical edges within WT GAL-7 and G16X mutants that support the importance of the dimer interface in allosteric communication. Indeed, the highest prevalent edges of the network are located at the dimer interface (Fig. 3). Interprotomer communication in WT GAL-7 is primarily formed by seven critical edges between protomers A and B: R20(A)-D103(B), R20(B)-D103(A), V18(A)-I91(B), V18(B)-I91(A), V18(A)-V18(B), F135(A)-V100(B), and F135(B)-V100(A). Except for the F135(A)-V100(B) and F135(B)-V100(A) pairs, these critical edges are conserved in the G16C and G16S network (Fig. 3*B*). These results indicate that only the F135(A)-V100(B) and F135(B)-V100(A) interactions are significantly weakened by the G16X mutations, and that the contact interactions involving other residues between protomers are similar or only slightly affected.

We further investigated the allosteric pathway between the two opposite GBSs within each protomer using the shortest path method, *i.e.*, the path for which the sum of its constituent edges is minimized. The shortest pathway connecting both opposite GBSs exhibits an identical pathway weight value of 0.27 and is identical for both WT and G16C: N62(A)-F61(A)-Y106(A)-A104(A)-D103(A)-R20(B)-V88(B)-F50(B)-H49(B) (Fig. 3*A*). The shortest pathway between the two G16S opposite GBSs also exhibits a similar weight value of 0.27 and involves the same sequence of residues, but transposed to the other protomer: N62(B)-F61(B)-Y106(B)-A104(B)-D103(B)-R20(A)-V88(A)-F50(A)-H49(A) (Fig. 3*A*). These results suggest that the primary interaction network connecting the GBS of each protomer is conserved in G16X mutants, although transposed between the A and B protomers in G16S.

## Conclusion

In this work, we used protein engineering to illuminate the importance of long-range interprotomer communication involving the homodimer interface in GAL-7. Our mutational results show that subtle interface perturbations can be exploited to alter residue communication between protomers, further supporting the previously observed positive cooperativity in GAL-7 (35). Subtle engineering changes that perturb dimer stability at the interface can be positively or negatively exploited to control the proapoptotic activity of GAL-7 at the cellular level. This functional modulation further demonstrates the relevance of this protein–protein interaction as an efficient interface for future rational drug discovery programs targeting GAL-7. Indeed, many structurally homologous galectins are involved in mediating subtle yet critical glycan-dependent and -independent interactions between pro- and antiapoptotic molecular partners in the cell (15, 33, 43). As a result of their highly homologous GBS interactions, the specific targeting of selected galectin members remains one of the most promising avenues for future disease treatments. Our results identified residues involved in dimer stability and allosteric communication between protomers, along with altered dynamic behaviors involving loops 1, 3, and 5, which could also potentially be used to modulate GAL-7 function. Overall, these observations highlight new avenues for the design of galectin-specific modulators to alter GAL-7-mediated functions in cancer and other diseases.

## Experimental procedures

### GAL-7 dimer stability assessment

The PoPMuSiC algorithm was used to estimate the impact of amino acid substitutions on protein stability (38). PoPMuSiC predicts changes in folding free energy (ΔΔG_F_) upon single-site mutation using solvent-accessibility-dependent combinations of statistical potentials. GAL-7 PDB entry 1BKZ was used as input for the algorithm, which requires the experimental or modeled 3D structure of the target protein. Analyses were also performed with the BeAtMuSiC algorithm (39), which uses similar potential combinations to predict changes in binding affinity (ΔΔG_B_) of protein–protein complexes upon mutation.

### DNA constructs and site-directed mutagenesis

The recombinant human gene encoding for galectin-7 (GAL-7) was subcloned into vector pET-22b(+) using *Nde*I and *Hin*dIII restriction enzymes and propagated as previously described (11). G16C and G16S mutants were generated with the Q5 Site-Directed Mutagenesis Kit (NEB) using the forward (G16Cf: 5′-atccgccct**tgc**acggtgctg-3′; G16Sf: 5′ atccgccct**tcc**acggtgctg-3′) and reverse (G16Xr: 5′-gccctcgggcagtgaggacttg-3′) primers. The F135S mutant was generated by the QuikChange site-directed mutagenesis method with PfU DNA polymerase (Bio Basic) using forward (F135f: 5′-gactccgtgaggatc **tcc**tgaaacgttgcgg-3′) and reverse (F135r: 5′-ccgcaacgtttca**gga**gatcctcacggagtc-3′) primers. All gene sequences were confirmed by DNA sequencing. Plasmid constructs were further employed for all protein expressions.

### Recombinant expression and purification of WT GAL-7 and variants

All pET-22b(+) constructs were transformed into *E. coli* BL21(DE3) for recombinant protein overexpression under control of the T7 promoter. A volume of 500 ml of lysogeny broth (LB) medium was inoculated with a 5-mL overnight preculture of *E. coli* BL21(DE3) carrying WT recombinant human GAL-7 or G16X mutant plasmids. Culture growth was carried out at 37 °C until OD_600nm_ = 0.6–0.7, after which protein expression was induced with 0.5 mM isopropyl-β-D-1-thiogalactopyranoside (IPTG) at 16 °C overnight. Bacterial cells were harvested by centrifugation for 30 min at 3800*g* (4 °C). Pellets were resuspended in 80 ml of buffer A (50 mM Tris pH 8.0, 150 mM NaCl) and lysed by sonication using a 1/2″ wave horn connected to a 450 Sonifier (Branson). Sonication was performed at power output level 7 with 70% pulse rate for 2-min cycles and 1 min cooling between each cycle. Cells were completely lysed after four cycles. The sonicated lysate was centrifuged for 30 min at 17,600*g* (4 °C). The supernatant was filtered and the protein was purified by lactose affinity gravity-flow purification at 4 °C. A 2-ml volume of α-lactose-agarose matrix (Sigma-Aldrich, Oakville, ON) was added into a 24 ml gravity column and then equilibrated with 50 ml buffer A. Filtered supernatant was applied to the column, which was then washed extensively with 50 ml buffer A. The pure protein was eluted with 15 ml buffer B (50 mM Tris pH 8.0, 150 mM NaCl, 150 mM α-lactose) and 1.5 ml fractions were collected. α-lactose was removed from the eluted protein by dilution in buffer C (20 mM Tris pH 8.0, 150 mM NaCl) for protein crystallization, buffer D (20 mM potassium phosphate, pH 7.2) for circular dichroism (CD) and isothermal titration calorimetry (ITC), or PBS (0.144 g/L KH_2_PO_4_, 0.795 g/l NaH_2_PO_4_, 9 g/l NaCl, pH 7.4) for microscale thermophoresis (MST) and cell assays. Dilution factors were set at 160,000-fold or higher to remove all α-lactose traces. Protein solutions were further concentrated using 3-kDa Amicon Ultra 15 ml Filters (EMD Millipore) at 3800*g* (4 °C).

### Translational diffusion analysis of free and lactose-bound WT GAL-7

Diffusion measurements were conducted using the BPP-LED (bipolar pulse pair–longitudinal-eddy-current delay) sequence (50), modified to include continuous wave water saturation during the relaxation delay, diffusion period, and LED period (*i.e.*, the ledbpgppr2s sequence as provided by the spectrometer vendor). In the BPP-LED experiment, the NMR signal intensity (I) is dependent on the molecular diffusion coefficient (D) and may be expressed as a function of the strength of the gradients used to probe the diffusion coefficient (g):

$$I\left(g\right)=I\left(0\right)\text{exp}\left[-D{\left(\gamma g\delta \right)}^{2}\left(\Delta -\frac{\delta }{3}-\frac{\tau }{2}\right)\right]$$

where γ is the ^1^H gyromagnetic ratio, δ/2 is the duration of each gradient pulse, Δ is the delay between the so-called “encoding” and “decoding” gradients, and τ is the gradient stabilization delay. In this work, δ/2 was fixed at 4.3 ms, Δ was held at 70 ms, and τ was maintained at 0.226 ms, while g was varied linearly in 40 steps from 3.6 to 32.5 G/cm (accounting for the sine-bell amplitude profile of the gradient pulses). The LED time was set to 5 ms. Sixteen transients were collected at each gradient strength, with a 5-s relaxation delay between scans, for a total experiment time of roughly 1 h. The sample temperature was regulated at 25 ± 1 °C, using a high gas flow of 1070 L/h to minimize convection-related artifacts in the diffusion measurement (51). Spectra were processed using a 20 Hz line-broadening window function along with polynomial baseline correction. Signal intensity of the protein in the methyl region (∼0.8 ppm) was integrated to provide 40 values of I(g). The methyl region was selected because it is far away from peaks associated with water or buffer, is less likely to be affected by solvent exchange, and is intense. A nonlinear-least-squares fit of the I(g) values against g provided the reported diffusion coefficients. The error in the diffusion measurement was calculated as 0.14 × 10^−11^ m^2^/s, as determined from the spread of fitted diffusion coefficients from five technical replicates of the diffusion measurement of the final titration point.

### Apoptosis assays with Annexin V/PI staining

Apoptosis was measured by flow cytometry using FITC-labeled Annexin V (Biolegend, San Diego, CA) and propidium iodide (PI) (34). Increasing concentrations of WT and GAL-7 variants (1–50 μM) were incubated with 2.5 × 10^5^ Jurkat T cells maintained in RPMI 1640 medium (Life Technologies, Burlington, ON) at 37 °C for 4 h. After incubation, cells were centrifuged for 8 min at 900*g* at 4 °C. Cell pellets were then resuspended in 100 μl of a solution containing Annexin V-FITC buffer (0.01 M HEPES pH 7.4, 0.14 M NaCl, 2.5 mM CaCl_2_, 0.63 μg/ml Annexin V) (Biolegend, San Diego, CA) and incubated for 15 min at room temperature in the dark. Four-hundred microliters of propidium iodide (PI) buffer (0.01 M HEPES pH 7.4, 0.14 M NaCl, 2.5 mM CaCl_2_, 0.25 μg/ml PI) was added to cells prior to flow cytometry analysis. In total, 5000 events were recorded and analyzed using a BD FACSCalibur flow cytometer. Controls included unstained cells to set positivity and a no-GAL-7 untreated control, value of which was subtracted for each data point. Results were based on three independent assays performed in duplicate. Proapoptotic activity was normalized to activity of 50 μM WT GAL-7 and evaluated the day of the assay, and the resulting percentages were plotted as a function of variant concentration using GraphPad Prism 9.0 (GraphPad Software). EC50 values were determined by nonlinear least squares regression fitting. Fitted concentration curves and best-fit values were compared using the extra sum-of-squares F-test method. The comparison analysis concluded that the preferred model was each dataset representing a different curve (a = 0.05, *p* ≤ 0.0003).

### Circular dichroism and thermal unfolding

Thermal unfolding of WT and GAL-7 variants was monitored by circular dichroism (CD) using a Jasco J-815 spectropolarimeter equipped with Peltier Jasco CDF-426S/15 thermostatic system. All thermal scanning experiments were acquired with 50 μM apo or holo protein (in presence of 6 mM α-lactose) in 200 μl buffer D (20 mM potassium phosphate, pH 7.2). Initial spectra were acquired at 20 °C from 250 nm to 200 nm in a 1 mm pathlength quartz cuvette. Thermal denaturation experiments were performed by monitoring changes in ellipticity at 220 nm between 20 °C and 80 °C with a heating rate of 1 °C/min. T_m_ values were determined using the first-order derivatives and polynomial functions of the Jasco Spectra Manager software with the Savitzky–Golay algorithm.

### Microscale thermophoresis (MST)

GAL-7 dimer equilibrium affinity was measured by MST using the Monolith NT.115 Pico instrument (NanoTemper Technologies GmbH) at 25 °C. In accordance with the manufacturer's protocol, WT GAL-7 and G16S variants were labeled with RED-NHS second generation using a Monolith NT.115 Protein Labeling Kit (Nanotemper Technologies) in PBS buffer. The excessive dye was separated by the provided column and protein was eluted in 20 mM Tris-HCl pH 8, 150 mM NaCl, supplemented with 0.05 % (v/v) Tween 20. Each binding assay experiment consisted of 16 2-fold serial dilutions of 50 μM (starting concentration) unlabeled GAL-7 prepared in 5 nM labeled GAL-7. All samples were incubated at 40 °C in a water bath for 45 min prior to loading into a NT.115 MST premium coated capillary. MST was induced by a 21s infrared laser (IR-laser) activation at 25 °C. All experiments were performed in triplicate. Raw data were preanalyzed and extracted using MO.Affinity software, version 2.3 (Nanotemper Technologies). Thermophoresis-induced changes in fluorescence were plotted as a function of unlabeled GAL-7 concentration using GraphPad Prism 9.0 (GraphPad Software). Dimer equilibrium dissociation constants (*K*_D_) were determined using the least squares regression fitting method. The G16S homodimer *K*_D_ was determined with the initial fluorescent signal while the WT homodimer *K*_D_ was calculated using the MST on-time signal between 4 and 5 s.

### Isothermal titration calorimetry (ITC)

All ITC experiments were carried out in triplicate at 25 °C using a Nano ITC microcalorimeter (TA Instruments). In total, 300 μl of 200 μM WT GAL-7 and G16X variants were prepared in 20 mM potassium phosphate (pH 7.2) and injected in the Nano ITC cell. 6 mM *α*-lactose was dissolved in the same buffer and filled in the syringe. Titration was performed with 25 injections of 2 μl ligand into protein with a stirring rate of 150 rpm and a 150-s interval between each injection. A blank experiment was carried out by titrating each ligand in a protein-free buffer. Data was analyzed and fitted using the NanoAnalyze software v2.3.6 (TA Instruments).

### Protein crystallization

#### Lactose-bound WT GAL-7

Cocrystallization of WT GAL-7 with α-lactose was performed using the sitting-drop vapor diffusion technique. Crystals were obtained by incubating a drop consisting of a 1:1 mixture of 1 μl of 7.5 mg/ml WT GAL-7 solution (50 mM Tris pH 8.0, 150 mM NaCl, 7.5 mM α-lactose) with 0.1 M NaCl, 0.1 M Tris pH 8.0, 20 % PEG 3350, 15% glycerol at room temperature after 1 week. Crystals were cryo-protected by soaking in a solution of 0.1 M NaCl, 0.1 M Tris pH 8.0, 20 % PEG 3350, 30% glycerol.

#### Apo GAL-7 (G16C)

One microliter of 7.5 mg/ml GAL-7 (G16C) solution (in buffer C) was mixed at 1:1 ratio with 0.1 M NaCl, 0.1 M Tris pH 8.0, 20 % PEG 3350, 15% glycerol. Crystals were obtained by the sitting-drop vapor diffusion technique at room temperature after 1 week. Crystals were cryo-protected by soaking in a solution of 0.1 M NaCl, 0.1 M Tris pH 8.0, 20 % PEG 3350, 30% glycerol.

#### Lactose-bound GAL-7 (G16C)

Cocrystallization of GAL-7 (G16C) with α-lactose was performed by the hanging-drop vapor diffusion technique. Crystals were obtained by incubating a drop consisting of a 1:1 mixture of 1 μl of 7.5 mg/ml GAL-7 (G16C) solution (50 mM Tris pH 8.0, 150 mM NaCl, 7.5 mM α-lactose) with 0.1 M NaCl, 0.1 M Tris pH 8.0, 25 % PEG 3350 at room temperature after 1 week. Crystals were cryo-protected by soaking in a solution of 0.1 M NaCl, 0.1 M Tris pH 8.0, 25 % PEG 3350, 30% glycerol.

#### Apo GAL-7 (G16S)

One microliter of 7.5 mg/ml GAL-7 (G16S) solution (in buffer C) was mixed at ratio 1:1 with 0.1 M NaCl, 0.1 M Tris pH 8.0, 20 % PEG 3350, 17.5% glycerol. Crystals were obtained by the sitting-drop vapor diffusion technique at room temperature after 1 week.

#### Lactose-bound GAL-7 (G16S)

Cocrystallization of GAL-7 (G16S) with α-lactose was performed by the hanging-drop vapor diffusion technique. Crystals were obtained by incubating a drop consisting of a 1:1 mixture of 1 μl of 7.5 mg/ml GAL-7 (G16S) solution (50 mM Tris pH 8.0, 150 mM NaCl, 150 mM α-lactose) with 0.1 M NaCl, 0.1 M Tris pH 8.0, 16 % PEG 3350 at room temperature after 1 week. Crystals were cryo-protected by soaking in a solution of 0.1 M NaCl, 0.1 M Tris pH 8.0, 15 % PEG 3350, 20% glycerol.

### Data collection, structure resolution, and refinement

All diffraction data of crystals in the presence and absence of α-lactose were collected at the Canadian Macromolecular Crystallography Facility Beamline 08B1-1 and 08ID-1 of the Canadian Light Source Synchrotron (52, 53). Raw data was immediately processed on the MxLIVE platform after collection. Structure resolution and refinement were carried out using the PHENIX software suite. Phase was calculated using the molecular replacement method using 1BKZ and 4GAL PDB structures as models for apo and holo structures, respectively. All structural comparisons and visualizations were performed with the Open-Source PyMOL Molecular Graphics System, Version 2.4 (Schrödinger, LLC). The Cα B-factor profiles were normalized and compared using the BANΔIT server using IBM z-Score (MADE) and MMLigner methods (54).

### Statistical analysis

Results represent at least three independent experiments and are plotted as mean with standard error of the mean (SEM) using GraphPad Prism 9.0 (GraphPad Software). Statistical significance was evaluated with *F-test* (cell assays, ITC and MST experiments) or one-way ANOVA with Tukey post-hoc tests (CD experiments). Two data sets were considered significantly different if *p*-value ≤ 0.05.

### Molecular dynamics simulations

Structural coordinates from PDB 4GAL (37) were used to build the WT GAL-7, G16C, and G16S apo systems. Ionizable residues were considered in their standard protonation state at pH 7.0 with neutral histidine protons placed at ND1 or NE2 positions, according to the interactions with their respective neighbors within the structure. Systems were built using the CHARMM-GUI (55, 56, 57) and the G16C and G16S mutations were introduced using the CHARMM-GUI tools. Structures were immersed in neutrally charged orthogonal boxes of water with a 10 Å distance from the protein to the edges of each box. Na^+^ and Cl^−^ ions were added at a concentration of 150 mM. MD simulations were performed with NAMD 2.13b1 (58) using the CHARMM36m force field parameters for proteins and carbohydrates (59) and TIP3P waters (60). Simulations were carried out at 303.15 K under isothermal-isobaric (NPT) ensemble conditions with a 2-fs time step and periodic boundary conditions. Langevin damping with a coefficient of 1 ps^−1^ was used to maintain constant temperature, while pressure was controlled by a Nosé–Hoover Langevin piston at 1 atm. Bond length between hydrogen and heavy atoms was constrained using SETTLE (61) for water molecules and SHAKE (62) for all other molecules. Cutoffs for the short-range electrostatics and the Lennard–Jones interactions were set at 12 Å, with the latter smoothed *via* a switching function over the range of 10–12 Å. Long-range electrostatic interactions were calculated with the Particle Mesh Ewald (PME) method (63, 64) using a sixth-order interpolation and a grid spacing of ≈1 Å at every integration step. Nonbonded pair lists were updated at every ten steps, and coordinates were saved every 10 ps for analysis. For each system (WT, G16C and G16S), three 1000-ns trajectories were recorded.

### Trajectory analysis

The first 500 ns of all trajectories was considered as equilibration time and was not included in the analyses. The last 500 ns of each of the trajectories was concatenated into three trajectories (one for each system) at a rate of ten frames every ns, for a total of 15,000 frames per trajectory. Only the proteins were included in the concatenated trajectories, which were aligned on the initial structure. The interface binding energies of WT, G16C, and G16S dimers were evaluated using the *AnalyseComplex* command from FoldX (44). This analysis was conducted on 5000 frames from the respective concatenated trajectories. For surface areas, final values were calculated using ten block averages over the trajectories and errors were calculated as standard deviations.

### Allosteric analysis

The protein dynamical network analysis was realized following the methodology exposed in a previous work (48). In short, the nodes of the network were represented by the residue heavy atom center of mass. Edges that transfer allosteric information between the nodes were drawn between the nodes for which the respective residue maintains any of its heavy atoms within a distance of 4.5 Å for at least 75% of the time in the trajectories. The weight of the edges between the nodes *i* and *j* was defined as the coefficient of variation of the distance between the nodes:

$$C_{ij}=\frac{\sqrt{\langle {\left(r_{ij}-\langle r_{ij}\rangle \right)}^{2}\rangle }}{\langle r_{ij}\rangle }$$

where *r*_*ij*_ = |r_*ij*_| = |r_*i*_ − r_*j*_| is the distance between the center of mass of residues *i* and *j*. In contrast to the calculation of the positional fluctuation correlations, the separation distance approach does not require prior removal of the global motions. NetworkX (http://conference.scipy.org/proceedings/scipy2008/paper_2) was used to calculate the edge betweenness centralities using the Ulrik-Brandes algorithm (65). All edges were ranked based on their betweenness centrality, the critical edges identified as edges with a prevalence of being part of an optimal path between any two nodes of at least three standard deviations (3σ) from the edge prevalence distribution.

## Data availability

X-ray coordinates for human GAL-7 in complex with 4-O-beta-D-galactopyranosyl-D-glucose, apo GAL-7 variant G16C, holo GAL-7 variant G16C in complex with lactose, apo GAL-7 variant G16S, and holo GAL-7 variant G16S in complex with lactose have been deposited in the RCSB PDB under accession codes 6VTO, 6VTP, 6VTQ, 6VTR, and 6VTS, respectively.

## Supporting information

This article contains supporting information.

## Conflict of interest

The authors declare that they have no conflicts of interest with the contents of this article.
