# Supplementary material for: Perturbing dimer interactions and allosteric communication modulates the immunosuppressive activity of human galectin-7
Source: J Biol Chem. 2021 Oct 19;297(5):101308. doi: 10.1016/j.jbc.2021.101308 (PMC8592873; doi:10.1016/j.jbc.2021.101308)
Supplement: Tables S1–S4 and Figures S1–S5 [file mmc1.docx]

SUPPORTING INFORMATION

Perturbing dimer interactions and allosteric communication modulates the immunosuppressive activity of human galectin-7

N. T. Hang Pham^1^, Myriam Létourneau^1^, Marlène Fortier^1^, Gabriel Bégin^2,5^, M. Sameer Al-Abdul-Wahid^3^, Fabrizio Pucci^4^, Benjamin Folch^1^, Marianne Rooman^4^, David Chatenet^1^, Yves St-Pierre^1^, Patrick Lagüe^2, 5^, Charles Calmettes^1,5^ & Nicolas Doucet^1,5*^

^1^Centre Armand-Frappier Santé Biotechnologie, Institut National de la Recherche Scientifique (INRS), Université du Québec, Laval, QC, Canada.

^2^Département de Biochimie, de Microbiologie et de Bio-informatique and Institut de Biologie Intégrative et des Systèmes (IBIS), Université Laval, Québec, Canada.

^3^Nuclear Magnetic Resonance Centre, University of Guelph, Guelph, ON, Canada.

^4^Computational Biology and Bioinformatics, Université Libre de Bruxelles, Brussels, Belgium.

^5^PROTEO, the Québec Network for Research on Protein Function, Engineering, and Applications, Université Laval, Québec, QC, Canada.

**Table S1.** List of mutations exhibiting the largest folding free energy difference (∆∆G_F_) between dimer (D) and monomer (M) forms of GAL-7, as predicted by PoPMuSiC (1). The change in binding affinity upon mutation (∆∆G_B_) was predicted by BeATMuSiC (2). Secondary structure elements turn (T), coil (C), and extended β-strand (E) were computed by an in-house program. The two mutations predicted to destabilize the dimer state relative to the monomeric state are shown in dark red and have been selected for further experimental characterization.

| **Posi-**  **tion** | **Wild-**  **type** | **Mu-tant** | **Secondary Structure** | **RSA(M) %** | **∆∆G_F_(M)**  **(kcal/mol)** | **RSA(D)**  **(%)** | **∆∆G_F_(D)**  **(kcal/mol)** | **∆∆G_F_(D)-**  **∆∆G_F_(M)** | **∆∆G_B_**  **(kcal/mol)** |
| --- | --- | --- | --- | --- | --- | --- | --- | --- | --- |
| 16 | GLY | GLU | T | 67 | 0.79 | 0 | 3.71 | 2.92 | 6.47 |
| 16 | GLY | ASP | T | 67 | 0.44 | 0 | 3.18 | 2.74 | 5.91 |
| 16 | GLY | LYS | T | 67 | 0.90 | 0 | 3.36 | 2.46 | 5.43 |
| **16** | **GLY** | **SER** | **T** | **67** | **0.50** | **0** | **2.64** | **2.14** | **4.36** |
| 16 | GLY | ASN | T | 67 | 0.29 | 0 | 2.37 | 2.08 | 4.06 |
| 16 | GLY | THR | T | 67 | 0.88 | 0 | 2.90 | 2.02 | 4.29 |
| 18 | VAL | GLU | E | 23 | 0.93 | 0 | 3.47 | 2.54 | 5.95 |
| 18 | VAL | LYS | E | 23 | 1.03 | 0 | 3.30 | 2.27 | 5.52 |
| 18 | VAL | ASP | E | 23 | 1.58 | 0 | 3.71 | 2.13 | 5.44 |
| 91 | ILE | GLU | E | 19 | 2.05 | 0 | 4.46 | 2.41 | 6.83 |
| 91 | ILE | LYS | E | 19 | 2.04 | 0 | 4.23 | 2.19 | 6.36 |
| 91 | ILE | ASP | E | 19 | 2.56 | 0 | 4.74 | 2.18 | 6.42 |
| 91 | ILE | PRO | E | 19 | 2.76 | 0 | 4.79 | 2.03 | 6.21 |
| 135 | PHE | GLY | C | 68 | 0.36 | 10 | 3.33 | 2.97 | 6.60 |
| 135 | PHE | ASP | C | 68 | 0.13 | 10 | 2.80 | 2.67 | 5.78 |
| **135** | **PHE** | **SER** | **C** | **68** | **0.20** | **10** | **2.84** | **2.64** | **5.66** |
| 135 | PHE | ALA | C | 68 | 0.28 | 10 | 2.91 | 2.63 | 5.60 |
| 135 | PHE | GLU | C | 68 | 0.07 | 10 | 2.51 | 2.44 | 5.08 |
| 135 | PHE | PRO | C | 68 | 0.25 | 10 | 2.64 | 2.39 | 5.13 |
| 135 | PHE | LYS | C | 68 | 0.16 | 10 | 2.50 | 2.34 | 4.98 |
| 135 | PHE | ASN | C | 68 | 0.16 | 10 | 2.49 | 2.33 | 4.93 |
| 135 | PHE | GLN | C | 68 | 0.12 | 10 | 2.38 | 2.26 | 4.70 |
| 135 | PHE | THR | C | 68 | 0.08 | 10 | 2.17 | 2.09 | 4.18 |
| 135 | PHE | CYS | C | 68 | 0.07 | 10 | 2.09 | 2.02 | 3.98 |

**Table S2.** ITC binding properties of α-lactose to WT GAL-7 and G16X variants

|  | **α-lactose** | | |
| --- | --- | --- | --- |
|  | **WT** | **G16C** | **G16S** |
| ***K*_D_** **(𝜇M)** | 135.3 ± 1.2 | 109.0 ± 1.0 | 122.7 ± 10.8 |
| **N** | 0.93 ± 0.03 | 0.93 ± 0.03 | 0.50 ± 0.01 |
| **Δ*H* (kJ/mol)** | -22.8 ± 0.1 | -22.4 ± 1.3 | -38.5 ± 6.9 |
| **Δ*S* (J/mol·K)** | -0.7 ± 3.2 | -0.5 ± 4.3 | -54.3 ± 23.9 |

**Table S3.** Crystallography data collection and refinement statistics.

|  | GAL-7 WT –  4-O-β-D-Galactopyranosyl-D-glucose | Apo GAL-7 G16C | GAL-7 G16C - Lactose | Apo GAL-7 G16S | GAL-7 G16S - Lactose |  |
| --- | --- | --- | --- | --- | --- | --- |
| **PDB ID** | 6VTO | 6VTP | 6VTQ | 6VTR | 6VTS |  |
| **Data collection^a^** | | | | | | |
| Space group | *P* 2_1_2_1_2_1_ | *P* 2_1_2_1_2_1_ | *P* 2_1_2_1_2_1_ | *P* 2_1_2_1_2_1_ | *P* 2_1_2_1_2_1_ |  |
| Cell dimensions | | | | | | |
| a, b, c (Å) | 30.52, 77.24, 111.44 | 53.60, 67.15, 71.92 | 30.37, 66.21, 111.56 | 54.01, 66.70, 71.43 | 30.30, 76.87, 113.77 |  |
| α, β, γ (°) | 90, 90, 90 | 90, 90, 90 | 90, 90, 90 | 90, 90, 90 | 90, 90, 90 |  |
| Resolution (Å) | 45.19 - 1.69  (1.75 - 1.69) | 42.98 - 2.3  (2.382 - 2.3) | 42.66 - 1.95  (2.02 - 1.95) | 48.75 - 2.3  (2.382 - 2.3) | 45.73 - 1.9  (1.968 - 1.9) |  |
| R_merge_ | 0.08592 (0.8361) | 0.1506 (2.416) | 0.1069 (1.037) | 0.04706 (0.4427) | 0.06688 (1.224) |  |
| I/σI | 15.71 (2.99) | 14.49 (1.21) | 16.73 (3.09) | 31.65 (5.86) | 23.53 (2.44) |  |
| CC_1/2_ | 0.999 (0.839) | 0.999 (0.569) | 0.999 (0.865) | 1 (0.964) | 1 (0.843) |  |
| CC* | 1 (0.955) | 1 (0.852) | 1 (0.963) | 1 (0.991) | 1 (0.957) |  |
| Completeness (%) | 99.09 (99.22) | 98.78 (97.62) | 99.86 (99.88) | 98.45 (97.48) | 99.84 (99.72) |  |
| Redundancy | 6.9 (7.2) | 9.7 (10.0) | 12.6 (12.7) | 9.8 (10.0) | 12.6 (12.4) |  |
| **Refinement** | | | | | | |
| Resolution (Å) | 1.69 | 2.3 | 1.95 | 2.3 | 1.9 |  |
| No. reflections | 30152 (2929) | 11899 (1148) | 17137 (1701) | 11780 (1161) | 21749 (2113) |  |
| R_work_ / R_free_ | 0.183/0.215 | 0.214/0.261 | 0.188/0.243 | 0.204/0.256 | 0.192/0.231 |  |
| No. of (non-hydrogen) atoms | 2502 | 2179 | 2250 | 2184 | 2266 |  |
| Protein | 2143 | 2123 | 2105 | 2115 | 2098 |  |
| Ligand/ion | 46 | 12 | 58 | 14 | 64 |  |
| Water | 313 | 44 | 87 | 55 | 104 |  |
| B factors | 19.73 | 65.11 | 33.28 | 60.72 | 38.32 |  |
| Protein | 18.2 | 65.53 | 33.15 | 61.11 | 37.81 |  |
| Ligand/ion | 24.17 | 48.07 | 34.46 | 53.9 | 45.53 |  |
| Water | 29.58 | 49.48 | 35.59 | 47.4 | 44.2 |  |
| r.m.s. deviations |  |  |  |  |  |  |
| Bond lengths (Å) | 0.012 | 0.005 | 0.013 | 0.004 | 0.011 |  |
| Bond angles (°) | 1.43 | 1.13 | 1.61 | 1.01 | 1.51 |  |

**Table S4.** Dimer interface surface area in apo WT and variants G16C and G16S.

|  | Surface area exposed to dimer interface (Å^2^)^a^ | | |
| --- | --- | --- | --- |
| Subunit | **WT** | **G16C** | **G16S** |
| A | 715 (8) | 661 (7) | 650 (5) |
| B | 613 (6) | 565 (6) | 558 (5) |

^a^The protein surface area exposed to the dimer interface was calculated using the *measure sasa* function of the VMD program (3) in WT, G16C and G16S, respectively. For each system, the surface of each residue exposed to the dimer interface was obtained from the difference in their solvent-accessible surface area (sasa) calculated from the dimer and their corresponding monomers. The total surface exposed to the interface from each monomer was obtained from the sum of the values from each residue composing each respective monomer. Final values were calculated using 10 block averages over the trajectories and errors (in parentheses) were calculated as standard deviations. Structural reorganization results in overall reduction of the surface area of the dimer interface in variants G16C and G16S.


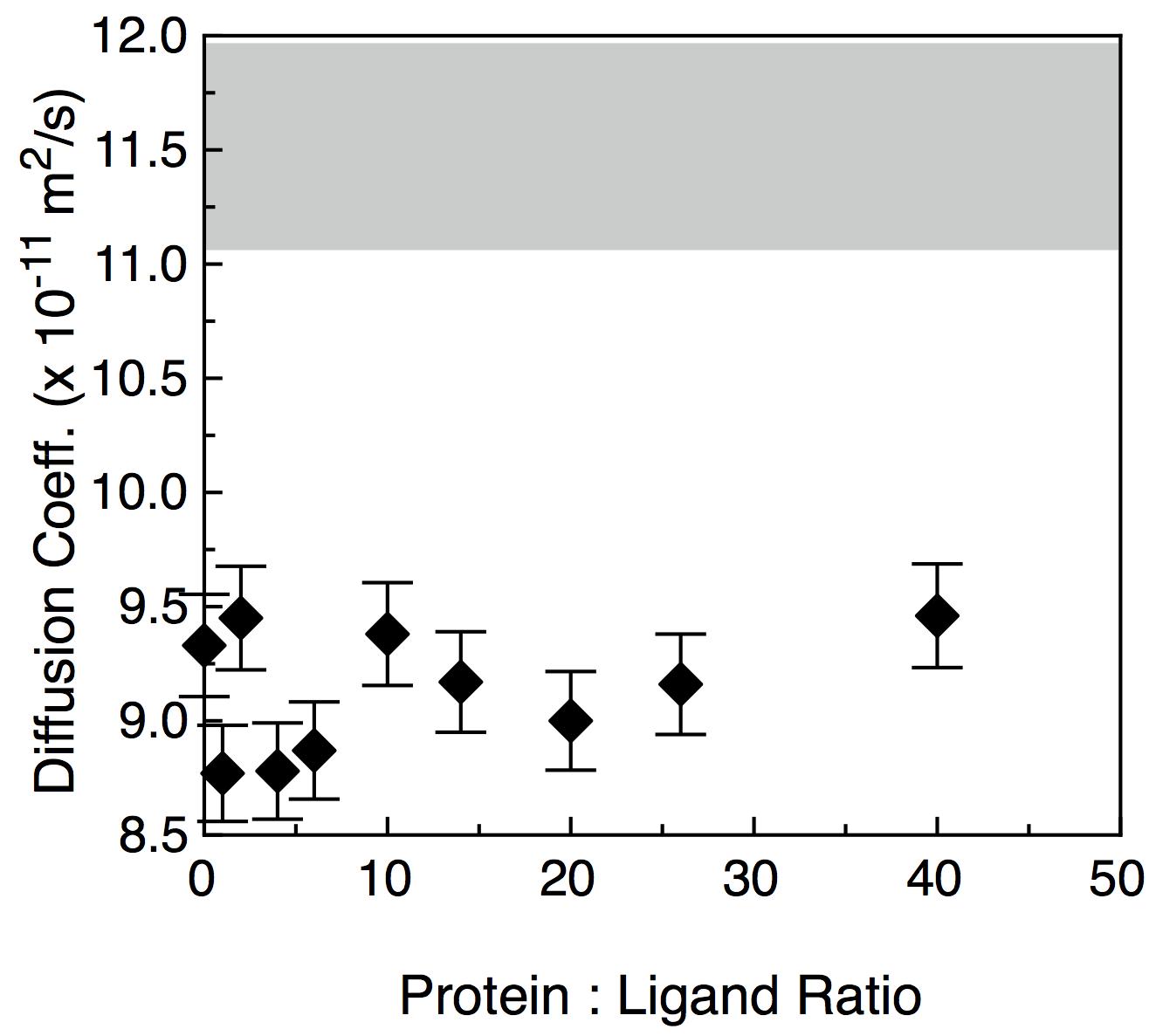


**Figure S1.** NMR extracted diffusion coefficients for WT GAL-7 in absence and presence of increasing concentrations of lactose. Increasing ligand-to-protein concentrations have a negligible effect on the NMR-extracted diffusion coefficients of GAL-7, suggesting that the protein exists as a homodimer in solution in absence or presence of lactose at the glycan binding site (GBS). Assuming the existence of a homodimeric GAL-7 state with diffusion coefficients roughly ranging between ~8.7 - 9.5 × 10^-11^ m^2^/s, the Stokes-Einstein equation would predict a diffusion coefficient ~26 % greater for a monomeric form of GAL-7. As a result, monomeric GAL-7 would appear in the upper shaded region of the graph. Diffusion measurements were conducted using the BPP-LED (bipolar pulse pair – longitudinal-eddy-current delay) sequence (4). See methods for details.

**
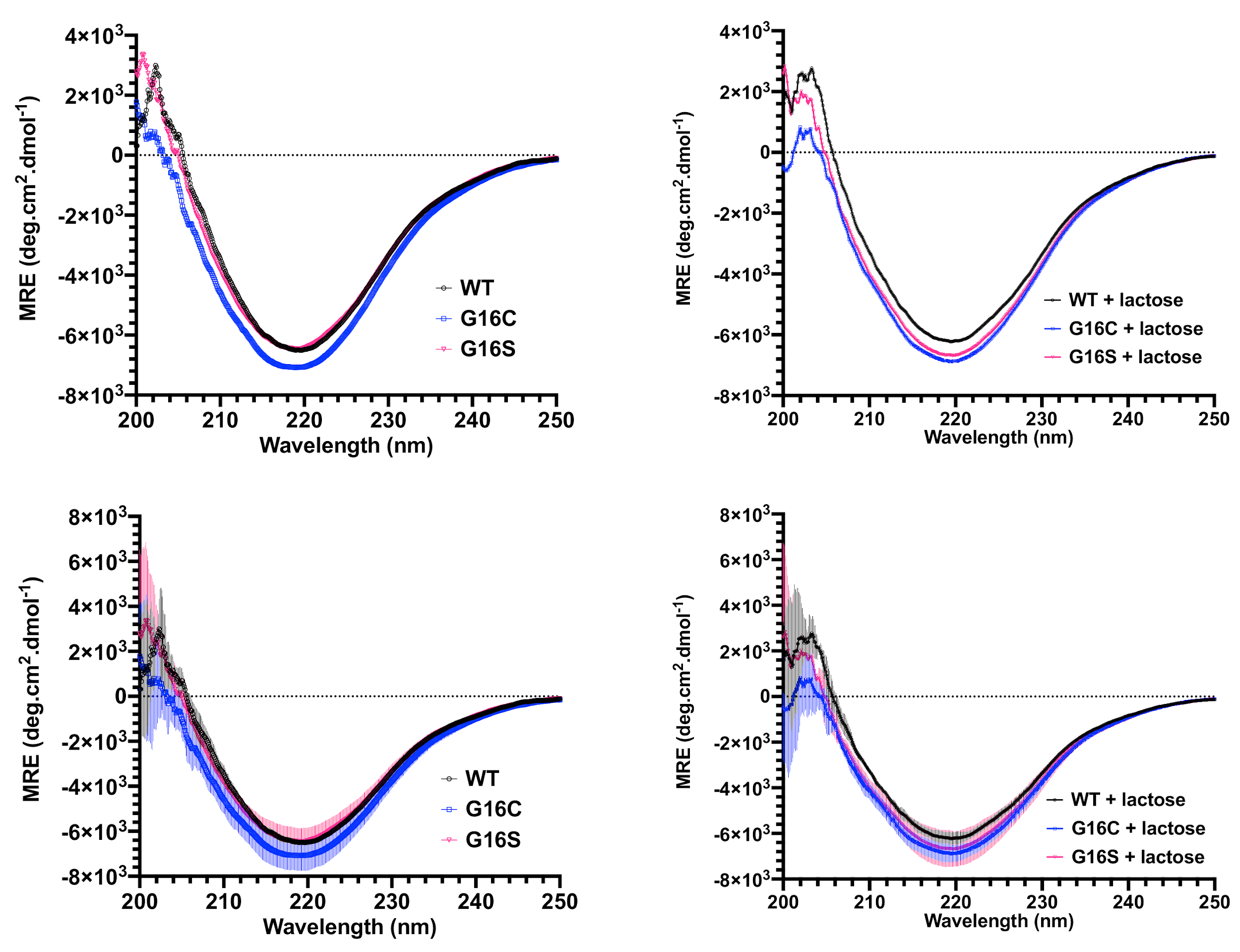
**

**Figure S2.** UV molar ellipticity spectra of WT GAL-7 (black), G16S (pink) and G16C (blue) obtained by CD spectropolarimetry. CD spectra are shown for apo proteins (left panels) and in presence of 6 mM α-lactose (right panels). Bottom panels show spectra with standard deviation.


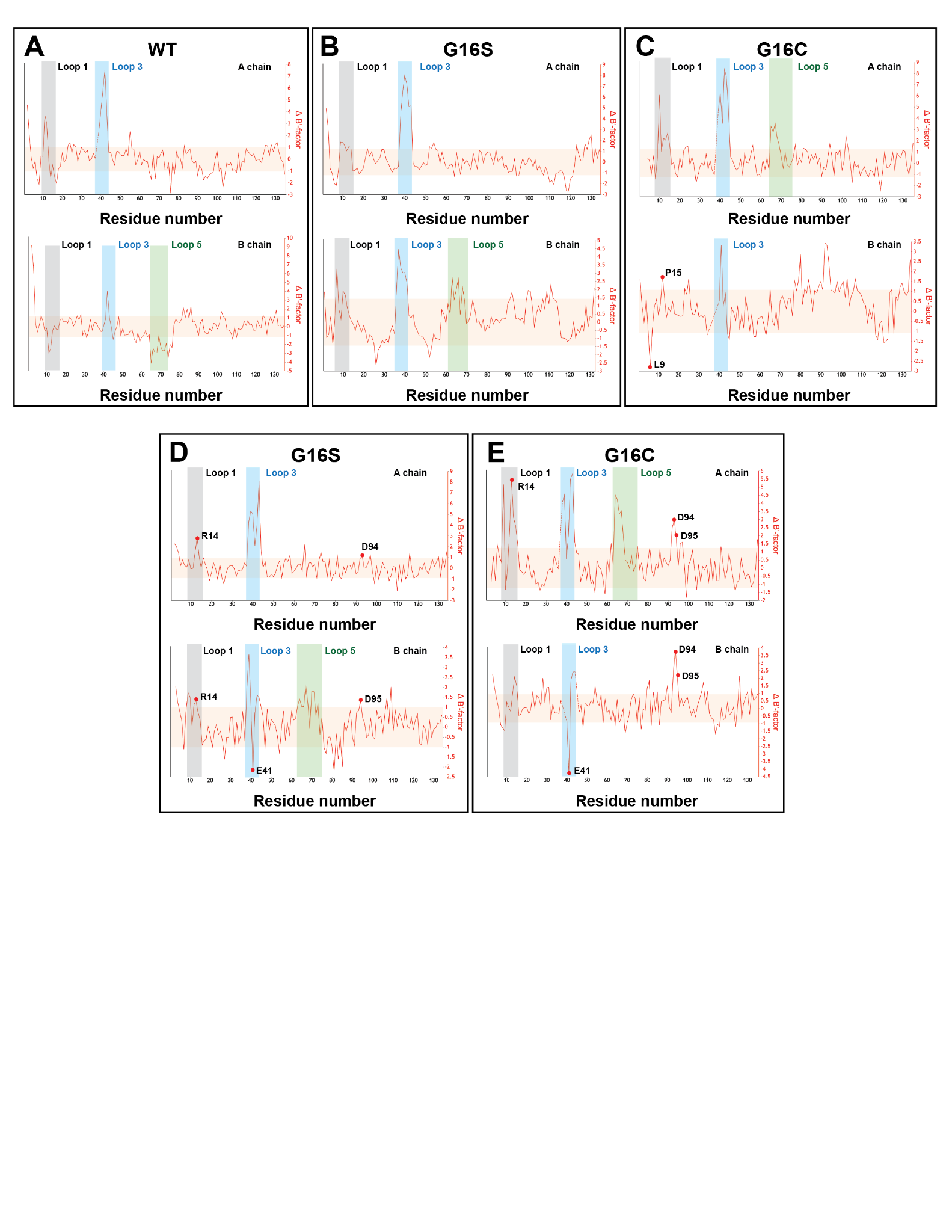


**Figure S3.** Plot of ∆B’-factor analysis (5) between WT, G16S and G16C variants of GAL-7. Cα B’-factor variations between apo *vs* holo structures for A) WT, B) G16S, and C) G16C. Cα ∆B’-factor values outside the orange box were found to be statistically significant (p<0.05). Residues 9-17 (loop 1), residues 39-43 (loop 3) and residues 64-74 (loop 5) are represented by grey, blue and green boxes, respectively. Chains A and B are plotted on top and bottom panels, respectively. Selected residues discussed in the text are labeled in black and shown as red dots on the plots. Cα B’-factor variations are also shown for D) holo WT *vs* holo G16S structures, and E) holo WT *vs* holo G16C structures. Positive ∆B’-factor values indicate increased flexibility and negative ∆B’-factor values indicate increased rigidification.


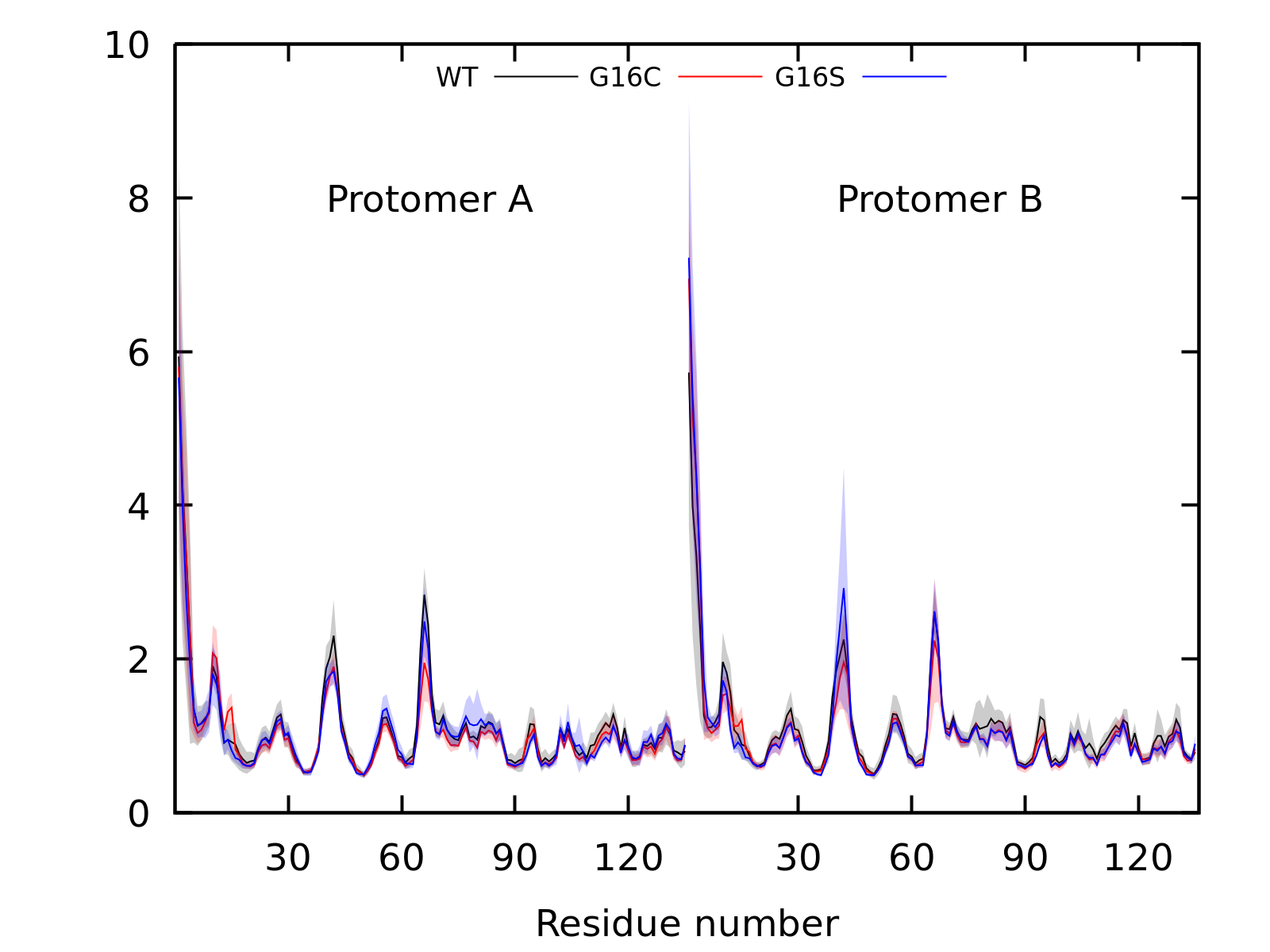


**Figure S4.** Plot of the backbone Cα atoms RMSFs for each residue of GAL-7 WT (black), G16C (blue) and G16S (red). The standard deviations calculated from the 10 block trajectories are represented by the shaded area.


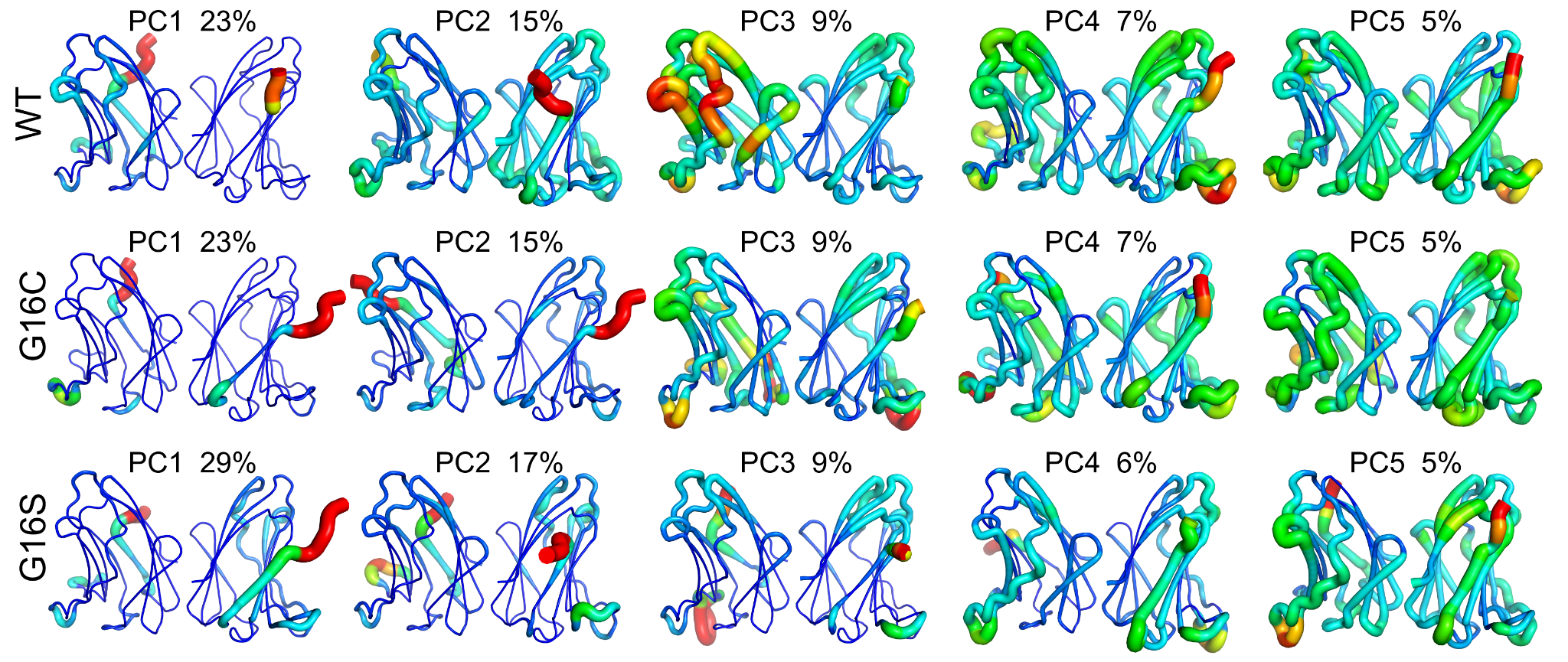


**Figure S5.** Average amplitude of motion for each residue involved in the first five PCA normal modes (PC1 to PC5) of WT (top), G16C (middle), and G16S (bottom). The size of the cartoon and the color spectrum correspond to the average amplitudes of motion within the first ten PCs. Width of putty cartoon representation and red color indicate greater amplitudes of motion. Percentages correspond to contributions to total motions calculated from the respective trajectories. The first ten PCA normal modes are presented in movies provided as supplementary material. All normal modes involve concerted motions between the two protomers. In agreement with the RMSF results for all 3 proteins, the first principal component (PC1, i.e. the greatest amplitude of motion) involves residues from the N-terminus as the primary concerted movement between protomers. The rocking motion observed between protomers as the principal concerted movement involves a pivot at the interface and is characterized by these motion amplitudes for most of the residues in the protein. Such movement appears in PC2 of WT GAL-7, and is also present for the remaining first five principal components. For G16C and G16S, rocking between the two protomers appears only in the third and fourth principal components, respectively. This movement is also present for the remaining first five principal components. Rocking movement between the two protomers as the main concerted motions accounts for 36% of the total motions in WT, while representing 21% and 11% in G16C and G16S, respectively. The concerted rocking movement between protomers is altered as a consequence of the single point mutation at the GAL-7 interface.

**References**

1. Gilis, D., and Rooman, M. (2000) PoPMuSiC, an algorithm for predicting protein mutant stability changes: application to prion proteins. *Protein Eng*. **13**, 849–856

2. Dehouck, Y., Kwasigroch, J. M., Rooman, M., and Gilis, D. (2013) BeAtMuSiC: Prediction of changes in protein-protein binding affinity on mutations. *Nucleic Acids Res*. **41**, W333-339

3. Humphrey, W., Dalke, A., and Schulten, K. (1996) VMD: visual molecular dynamics. *J Mol Graph*. **14**, 33–38, 27–28

4. Wu, D. H., Chen, A. D., and Johnson, C. S. (1995) An improved diffusion-ordered spectroscopy experiment incorporating bipolar-gradient pulses. *Journal of Magnetic Resonance, Series A*. **115**, 260–264

5. Barthels, F., Schirmeister, T., and Kersten, C. (2021) BANΔIT: B’-Factor Analysis for Drug Design and Structural Biology. *Molecular Informatics*. **40**, 2000144
